# Pharmacokinetics and safety of a fixed-dose combination of pregabalin and thioctic acid in healthy volunteers: a randomized, open-label, phase 1 crossover study

**DOI:** 10.3389/fphar.2025.1692519

**Published:** 2025-09-26

**Authors:** Edith Zárate, Cecilia Bravo-Lamicq, Diego Antonio Ocampo-Gutiérrez de Velasco, Oscar Arias-Carrión

**Affiliations:** ^1^ Psicofarma S.A. de C.V., Mexico City, Mexico; ^2^ División de Neurociencias Clínica, Instituto Nacional de Rehabilitación Luis Guillermo Ibarra Ibarra, Mexico City, Mexico; ^3^ Tecnológico de Monterrey, Escuela de Medicina y Ciencias de la Salud, Mexico City, Mexico

**Keywords:** pregabalin, alpha-lipoic acid, diabetic neuropathy, pharmacokinetics, fixed-dosecombination, phase 1 trial, UPLC-MS/MS, bioequivalence

## Abstract

**Background:**

Pregabalin (PGB) is a first-line therapy for painful diabetic neuropathy (PDN), but its adverse effects limit use. Thioctic acid, also known as α-lipoic acid (ALA), exhibits antioxidant and neuroprotective properties. The pharmacokinetic interaction profile of their combination in humans remains incompletely characterized.

**Methods:**

In this randomized, open-label, three-period, six-sequence, single-dose crossover study, 24 healthy adults (12 males, 12 females) received PGB (80 mg), ALA (400 mg), or their fixed-dose combination under fasting conditions, with 7-day washouts. Plasma concentrations were measured at 17 time points over 36 h using a validated UPLC–MS/MS method. Non-compartmental analysis was used to derive pharmacokinetic parameters (C_max_, t_max_, AUC_last_, AUC_inf_, t_½_). Bioequivalence was assessed using geometric mean ratios (GMRs) and 90% confidence intervals (CIs).

**Results:**

For PGB, the GMRs (90% CIs) for C_max_ and AUC_last_ were 91.2% (82.5%–100.8%) and 90.4% (80.9%–100.9%), respectively. For ALA, the GMRs were 108.9% (90.6%–130.8%) for C_max_ and 96.5% (87.7%–106.3%) for AUC_last_. All 90% CIs were within predefined bioequivalence ranges (80%–125% for PGB and ALA AUC_last_, 75%–133% for ALA C_max_). Adverse events were mild, transient, and resolved without intervention; no serious adverse events occurred.

**Conclusion:**

In this single-dose, Phase 1 trial in healthy volunteers, co-administration of PGB and ALA did not result in clinically significant pharmacokinetic interactions and was well tolerated. These findings provide preliminary pharmacokinetic evidence to support further clinical evaluation in patients with PDN.

## 1 Introduction

Painful diabetic neuropathy (PDN) is a frequent and disabling complication of diabetes mellitus, affecting up to half of patients with diabetic peripheral neuropathy and substantially impairing quality of life (Jensen et al., 2021; Pop-Busui et al., 2022). Despite several approved treatments, including pregabalin (PGB), duloxetine, and tapentadol, therapeutic responses are often suboptimal and long-term use is limited by adverse effects (D'Souza et al., 2022; Yeung et al., 2024).

PGB, a calcium channel α2δ ligand, has demonstrated efficacy in reducing pain intensity and improving sleep and daily function in neuropathic pain syndromes (Arezzo et al., 2008; Guan et al., 2011; Derry et al., 2019). Compared with tricyclic antidepressants, it offers a more favorable safety profile (Bansal et al., 2009). However, its dose-dependent adverse events, such as dizziness and somnolence, often compromise tolerability, particularly in older adults or those with comorbidities (Zaccara et al., 2011).

Thioctic acid (α-lipoic acid, ALA) is an endogenous antioxidant with neuroprotective properties that has been studied as adjunctive therapy for PDN. Intravenous ALA shows consistent benefit, while oral formulations present heterogeneous outcomes, partly due to high pharmacokinetic variability (Mijnhout et al., 2012; Papanas and Ziegler, 2014). Nevertheless, oral ALA at a dose of 600 mg/day has demonstrated improvements in sensory and motor symptoms with acceptable safety (Hsieh et al., 2023; Das et al., 2024; Mangarov et al., 2025). Combining PGB and ALA is hypothesized to provide complementary benefits, with symptom relief provided by PGB and pathophysiological modulation achieved through ALA (Yorek, 2024).

Clinical investigations suggest that this combination may enhance analgesic efficacy while allowing for reduced pregabalin dosing, potentially mitigating adverse events. Notably, a randomized controlled trial found that the combination of ALA and low-dose pregabalin outperformed monotherapy in terms of pain control and quality of life improvement, with fewer treatment discontinuations due to side effects (Park et al., 2022). However, contrasting results from the PAIN-CARE trial reported no superiority of the combination over pregabalin alone in pain reduction (Gilron et al., 2024), underscoring the need for pharmacokinetic and pharmacodynamic evaluations to clarify potential interactions.

Preclinical studies further support this hypothesis. Cruz-Álvarez et al. (2018) reported additive antiallodynic effects of pregabalin and ALA in a rat model of streptozotocin-induced PDN, while a more recent study using a spinal nerve ligation model demonstrated synergistic interaction, with isobolographic analysis confirming enhanced efficacy at reduced doses (γ = 0.524; 95% CI: 0.41–0.66; *p* < 0.05) (Zárate et al., 2025). These findings support the biological plausibility of combined therapy and highlight its potential to enhance efficacy and reduce adverse effects.

Human data on pharmacokinetic interactions between PGB and ALA remain limited. A previous study found no relevant interaction under multiple-dose administration (Rhee et al., 2018), but the absorption kinetics of a single-dose co-formulation had not been evaluated. To address this gap, we conducted a randomized, single-dose, crossover trial in healthy volunteers under fasting conditions. A single-dose design was chosen to minimize confounders from steady-state kinetics and to enable a precise assessment of absorption and elimination parameters in an early-phase setting. This study aimed to determine whether co-administration alters the pharmacokinetics of either agent at clinically relevant doses and to provide preliminary evidence for subsequent trials in PDN.

## 2 Methods

### 2.1 Study design

This randomized, open-label, single-dose, three-period, six-sequence crossover trial evaluated potential pharmacokinetic interactions between pregabalin and thioctic acid in healthy volunteers. The study complied with the Declaration of Helsinki and the International Council for Harmonisation Good Clinical Practice (ICH-GCP) guidelines. Regulatory approval was obtained from the Comisión Federal para la Protección contra Riesgos Sanitarios (COFEPRIS; authorization number 163300410B0341/2016, issued on October 18, 2016). Ethical approval was granted by the institutional research ethics committee (approval code PRO-142/16, dated July 29 2016). The trial was conducted at the Núcleo Clínico de Bioequivalencia, S.A. de C.V. (Cipriano Campos Alatorre No. 994, Col. Villas de Nilo, C.P. 44824, Guadalajara, Jalisco, México). Written informed consent was obtained from all participants prior to any study-related procedure.

Participants were randomized into one of six treatment sequences—ABC, BCA, CAB, BAC, CBA or ACB—each consisting of three treatment periods separated by a minimum of 7 days’ washout to prevent carryover effects. The interventions were as follows: Randomization sequences were generated using computer-based allocation, with concealment ensured through the use of sealed, opaque envelopes to minimize bias. Participants received capsules of either the active drug or a placebo, which were identical in appearance to maintain blinding and allocation concealment.

• A: Single oral dose of pregabalin (80 mg)
• B: Single oral dose of thioctic acid (400 mg)
• C: Fixed-dose combination of pregabalin (80 mg) and thioctic acid (400 mg)

All treatments were administered under fasting conditions, with participants refraining from food intake for at least 10 h prior to dosing and for 4 h afterwards to standardize absorption kinetics across treatment arms. The synergistic combination of pregabalin and α-lipoic acid for neuropathic pain is protected under patent family MX392835B (México), US12029727B2 (United States), and CA3047077C (Canada); EP3593795A4 (Europe) remains under examination, with coverage valid at least until December 16, 2036.

### 2.2 Study population

Healthy adult volunteers aged 18–50 years with a body mass index (BMI) between 18 and 27 kg/m^2^ were enrolled. Inclusion criteria required good general health, as determined by clinical history, physical examination, electrocardiogram, and standard laboratory tests. Participants were excluded if they had a known hypersensitivity to pregabalin or thioctic acid, pregnancy, a history of alcohol or drug abuse, or any chronic medical condition, especially hepatic, renal, cardiovascular, gastrointestinal, or neurologic disorders.

### 2.3 Pharmacokinetic sampling

Venous blood samples were drawn at 17 time points post-dose: 0 (pre-dose), 0.16, 0.33, 0.5, 0.75, 1, 1.25, 1.5, 2, 2.25, 2.5, 3, 6, 12, 24, and 36 h. Samples were collected into EDTA tubes, immediately centrifuged at 4000 rpm for 5 min at 4 °C, and plasma was separated and stored at −70 °C until assay. This sampling schedule enabled the capture of complete absorption and elimination profiles for both drugs.

### 2.4 Bioanalytical procedures

Plasma concentrations of PGB and ALA were quantified using a validated ultra-performance liquid chromatography–tandem mass spectrometry (UPLC–MS/MS) method, in accordance with the Mexican regulatory standard NOM-177-SSA1-2013. Briefly, 200 µL of plasma were precipitated with 1.5 mL of cold acetonitrile containing losartan (100 μg/mL) as the internal standard. Following centrifugation (4000 rpm, 10 min, 6 °C) and filtration, samples were injected into an Acquity UPLC HSS T3 column (100 mm × 2.1 mm, 1.7 µm) at 30 °C. The mobile phase consisted of 0.01 M ammonium acetate in 0.1% acetic acid and acetonitrile (20:80, v/v), delivered at a flow rate of 0.15 mL/min. Detection was performed in multiple reaction monitoring (MRM) mode, using positive ionization for pregabalin (m/z 160.2 → 142.0) and negative ionization for ALA (m/z 204.9 → 171.2). The method was linear over the ranges of 25–2000 ng/mL (PGB) and 20–10,000 ng/mL (ALA), with validated precision, accuracy, and stability under standard storage and processing conditions.

### 2.5 Pharmacokinetic and statistical analysis

Pharmacokinetic (PK) parameters were estimated using non-compartmental analysis. The primary PK variables included maximum plasma concentration (C_max_), time to C_max_ (t_max_), area under the plasma concentration–time curve to the last quantifiable concentration (AUC_last_), area under the curve extrapolated to infinity (AUC_inf_), and terminal elimination half-life (t_½_).

Descriptive statistics (mean, standard deviation) were calculated for all PK parameters. Bioequivalence between treatments was evaluated by calculating the geometric mean ratios (GMRs) and 90% confidence intervals (CIs) for the log-transformed maximum concentration (Cmax) and area under the curve last time point (AUClast). Acceptance criteria were set at 80%–125% for pregabalin and at 75%–133% for the Cmax of thioctic acid, due to its high intra-subject variability, consistent with prior reports (Rhee et al., 2018; Bulut et al., 2021).

A linear mixed-effects model appropriate for a 3-treatment, 3-period, 6-sequence crossover design was used to assess the fixed effects of sequence, period, and treatment, with subject nested within sequence as a random effect, in accordance with current regulatory guidelines (FDA (2022) and NOM-177-SSA1-2013). From this model, intra-subject and inter-subject coefficients of variation (CV%) were calculated for C_max_ and AUC_last_. Bioequivalence was further evaluated using the two one-sided t-test (TOST) procedure with a significance level of α = 0.05. All statistical analyses were performed using RStudio (version 2025.05.1) running R (version 4.5.1).

### 2.6 Sample size

The intra-subject coefficients of variation (18% for Cmax, 4.42% for AUC) were obtained from an internal pilot study conducted by our group. The sample size was estimated based on the statistical principles outlined by Chow and Wang (2001) for crossover bioequivalence trials, using the PowerTOST package in R. The calculation incorporated intra-individual coefficients of variation of 18% for C_max_ and 4.42% for AUC, obtained from a prior pilot study. Assuming a 5% difference between formulations (GMR = 0.95), a statistical power of 80%, and a 20% dropout rate, the required sample size was 23 subjects. To allow for equal gender representation, a final sample of 24 participants was planned.

## 3 Results

### 3.1 Participant characteristics

A total of 24 healthy adult volunteers (12 males and 12 females) were enrolled in the pharmacokinetic study. Baseline demographic data, including age, sex, weight, height, and body mass index (BMI), were collected (Table 1). The mean age was 29.5 ± 9.0 years, and the mean BMI was 23.4 ± 1.9 kg/m^2^. All participants except one completed the full crossover sequence and were included in the pharmacokinetic and safety analyses. Subject 19 was withdrawn after failing to attend the second study period due to reasons unrelated to the trial.

**TABLE 1:** Demographic data and sequence of administration of Pregabalin (A), Thioctic Acid (B), and the Fixed-Dose Combination (C).

Volunteer	Sex	Age (y)	Weight (kg)	Height (m)	BMI (kg/m^2^)	Sequence
1	M	37	64.5	1.62	24.58	ABC
2	M	47	82.0	1.76	26.47	BCA
3	M	30	81.5	1.88	23.06	CAB
4	F	35	68.5	1.60	26.76	ABC
5	M	27	70.0	1.68	24.80	BCA
6	M	23	61.5	1.62	23.43	ABC
7	F	43	58.5	1.53	24.99	CAB
8	F	45	60.5	1.60	23.63	BCA
9	M	26	60.5	1.73	20.21	CAB
10	F	40	63.2	1.55	26.31	BAC
11	F	22	74.5	1.76	24.05	CBA
12	F	25	57.0	1.63	21.45	ACB
13	M	26	64.0	1.71	21.89	CBA
14	M	28	72.0	1.76	23.24	BAC
15	F	20	51.0	1.55	21.23	ABC
16	F	20	66.9	1.60	26.13	CAB
17	M	43	63.0	1.60	24.61	ACB
18	F	26	51.0	1.52	22.07	CBA
19^a^	M	23	69.5	1.70	24.05	BAC
20	F	34	63.5	1.64	23.61	ACB
21	F	19	61.0	1.68	21.61	BCA
22	M	20	65.0	1.76	20.98	CBA
23	M	32	58.5	1.68	20.73	ACB
24	F	18	60.0	1.64	22.31	BAC
Mean		29.54	64.48	1.66	23.43	
SD		8.98	7.80	0.09	1.92	
Min		18	51.00	1.52	20.21	
Max		47	82.00	1.88	26.76	

^a^ Data correspond to all 24 randomized participants; subject 19 withdrew before completing the second period, resulting in 23 participants included in the pharmacokinetic and safety analyses.

### 3.2 Pharmacokinetic profiles

**FIGURE 1:**
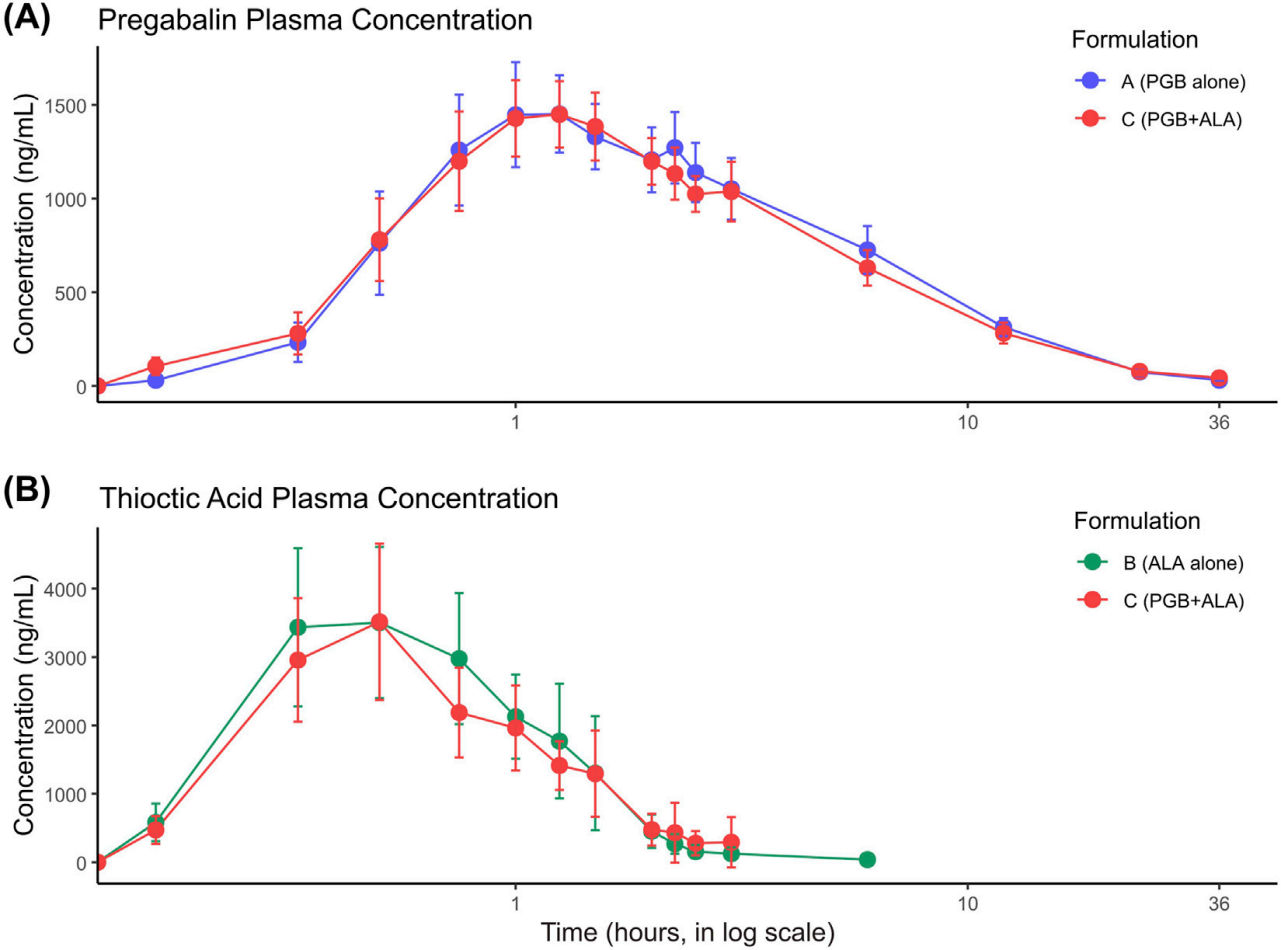
Plasma concentration–time profiles for pregabalin (PGB) and thioctic acid (ALA) following administration as monotherapy or in combination. **(A)** Mean plasma concentrations of PGB (ng/mL) up to 36 h after single-dose oral administration of PGB alone (80 mg, blue) or in fixed-dose combination with ALA (80 mg PGB + 400 mg ALA, red) in healthy volunteers (n = 23). Both formulations demonstrated rapid absorption, with peak concentrations achieved between 1 and 2 h post-dose, followed by a biphasic decline consistent with distribution and elimination phases. **(B)** Mean plasma concentrations of ALA (ng/mL) up to 6 h after single-dose oral administration of ALA alone (400 mg, green) or co-administered with PGB (red). ALA reached peak concentrations within the first hour in both formulations, reflecting its known rapid absorption and high variability. Values are presented as mean ± 95% confidence intervals (CI). The x-axis (time) is displayed on a logarithmic scale to enhance the visualization of early absorption kinetics. Supplementary figures display individual plasma concentration–time curves for both compounds, illustrating the degree of inter-individual variability observed, particularly for ALA.

Mean plasma concentration–time curves for pregabalin and thioctic acid following administration of each treatment are shown in Figure 1. For pregabalin, peak plasma concentrations were reached at approximately 1.4 h after administration. When co-administered with thioctic acid, pregabalin exhibited a slight reduction in C_max_ and overall exposure. Thioctic acid reached peak concentrations more rapidly, around 0.75 h post-dose, with minor differences between monotherapy and combination administration.

The non-compartmental pharmacokinetic parameters of pregabalin and thioctic acid are summarized in Table 2. When administered as a fixed-dose combination, pregabalin showed a modest decrease in C_max_ (1716.09 ± 425.67 ng/mL vs. 1839.57 ± 326.89 ng/mL) and AUC_last_ (10584.79 ± 3628.69 ng·h/mL vs. 11453.79 ± 2885.02 ng·h/mL) compared to pregabalin alone. The t_max_ and t_½_ values were similar across treatments. For thioctic acid, co-administration with pregabalin resulted in a slight reduction in AUClast (3625.76 ± 807.72 ng·h/mL vs. 3971.10 ± 1725.52 ng·h/mL) and a minimal change in C_max_ (5033.48 ± 2005.74 ng/mL vs. 5146.09 ± 2742.76 ng/mL). The t_max_ remained comparable between treatments.

**TABLE 2:** Non-compartmental pharmacokinetic parameters of pregabalin and thioctic acid after single-dose oral administration in monotherapy and fixed-dose combination (n = 23).

Parameter	Alone (mean ± SD)	Combination (mean ± SD)
Pregabalin		
C_max_ (ng/mL)	1839.57 ± 326.89	1716.09 ± 425.67
t_max_ (h)	1.41 ± 0.61	1.32 ± 0.56
AUC_last_ (ng·h/mL)	11453.79 ± 2885.02	10584.79 ± 3628.69
AUC_inf_ (ng·h/mL)	12029.85 ± 3316.38	11168.39 ± 3560.79
t_1/2_ (h)	5.72 ± 1.28	5.83 ± 1.41
Thioctic Acid		
C_max_ (ng/mL)	5146.09 ± 2742.76	5033.48 ± 2005.74
t_max_ (h)	0.75 ± 0.45	0.79 ± 0.53
AUC_last_ (ng·h/mL)	3971.10 ± 1725.52	3625.76 ± 807.72
AUC_inf_ (ng·h/mL)	4048.63 ± 1696.50	3653.00 ± 829.94
t_1/2_ (h)	0.37 ± 0.21	0.34 ± 0.15

C_max_: maximum observed plasma concentration; t_max_: time to reach C_max_; AUC_last_: area under the plasma concentration–time curve from time zero to the last measurable concentration; AUC_inf_: AUC, extrapolated to infinity; t_½_: terminal elimination half-life.

### 3.3 Bioequivalence evaluation

**FIGURE 2:**
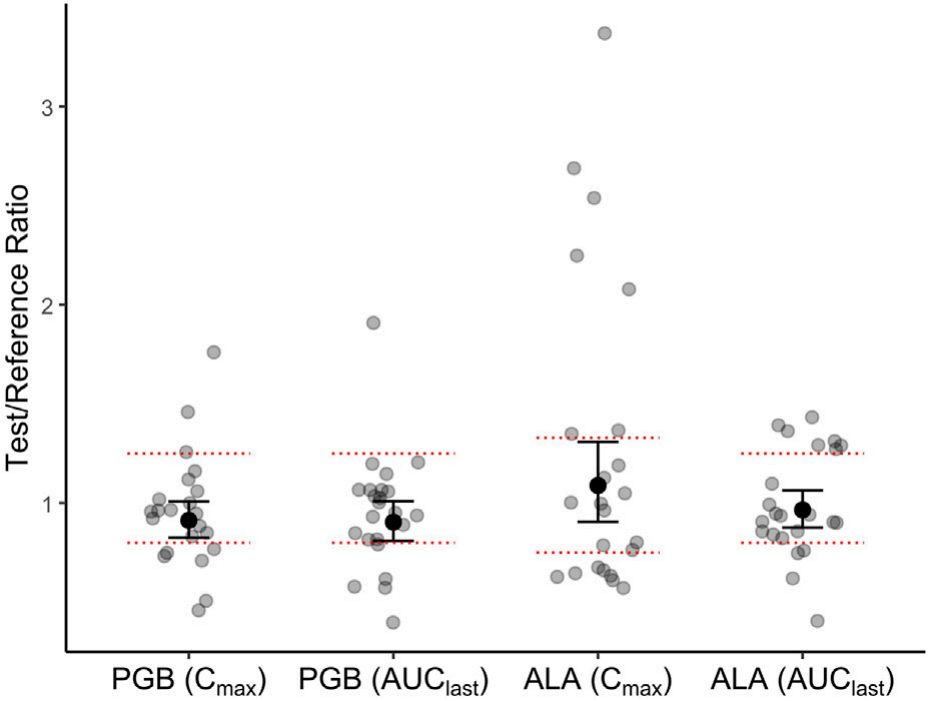
Individual participant ratios of test/reference treatments for maximum plasma concentration (Cmax) and area under the plasma concentration–time curve to the last measurable concentration (AUClast). Grey dots represent individual subject values; black dots represent the geometric mean ratios (GMRs), and vertical bars denote 90% confidence intervals (CIs). Data are shown for pregabalin (80 mg) and thioctic acid (400 mg) following single-dose administration alone and in fixed-dose combination in healthy volunteers (n = 23). The predefined no-effect (bioequivalence) boundaries are indicated by horizontal red dashed lines: 80%–125% for pregabalin and for AUClast of thioctic acid, and 75%–133% for Cmax of thioctic acid. These acceptance ranges follow regulatory guidance for highly variable drugs. All pharmacokinetic parameters are expressed in ng/mL.

Inter-individual variability was modest for pregabalin but notably higher for ALA, especially in Cmax, which has implications for clinical predictability. The GMRs of C_max_ and AUC_last_ for the fixed-dose combination (test) versus pregabalin or thioctic acid alone (reference), along with their 90% confidence intervals (CIs), are presented in Table 3 and illustrated in Figure 2. For pregabalin, the GMRs for C_max_ and AUC_last_ were 91.2% and 90.4%, respectively, with both CIs falling within the predefined bioequivalence range of 80%–125%. For thioctic acid, the GMR for AUC_last_ was 96.53%, with CIs within the standard range, while the GMR for C_max_ was 108.85%, remaining within the widened acceptance interval of 75%–133%. These results indicate no clinically significant pharmacokinetic interaction between pregabalin and thioctic acid.

**TABLE 3:** Geometric mean ratios (test/reference), 90% confidence intervals, and coefficients of variation for C_max_ and AUC_last_ of pregabalin and thioctic acid.

Parameter	Ratio (%)	90% CI	CV (%)	
			Intra-subject	Inter-subject
Pregabalin				
C_max_	91.20	82.51–100.81	19.84	13.45
AUC_last_	90.35	80.86–100.94	22.02	19.14
Thioctic Acid				
C_max_	108.85	90.57–130.82	37.29	34.47
AUC_last_	96.53	87.65–106.31	19.1	25.59

Bioequivalence acceptance ranges: 80%–125% for pregabalin and for AUC, of thioctic acid; 75%–133% for C_max_ of thioctic acid. Coefficients of variation (CV%) were estimated from the linear mixed-effects model.

Intra-subject and inter-subject coefficients of variation (CV%) are also shown in Table 3. Thioctic acid exhibited notably higher variability compared to pregabalin, particularly in C_max_.

Type III ANOVA using a linear mixed-effects model showed no statistically significant effects of sequence, period, or treatment on the log-transformed C_max_ and AUC_last_ values for either drug (p > 0.05 for all comparisons), indicating the absence of carryover effects and suggesting consistent pharmacokinetic behavior across treatment periods and sequences.

No significant difference in t_max_ was observed between the combination and monotherapy treatments for either pregabalin (p = 0.4661) or thioctic acid (p = 0.8765), as assessed by the Wilcoxon signed-rank test, indicating that co-administration did not alter the rate of absorption.

### 3.4 Safety and tolerability

A total of 18 adverse events (AEs) were reported across the three treatment arms. All AEs were classified as mild in intensity and resolved spontaneously without the need for medical intervention. No serious adverse events (SAEs) or study withdrawals due to AEs occurred.

For pregabalin alone, 8 AEs were reported in 7 subjects, with the most frequent being dizziness (6 events), followed by drowsiness and blurred vision. Thioctic acid alone was associated with 3 AEs in 3 subjects, including drowsiness, headache, and one case of mild vomiting. The fixed-dose combination led to 7 AEs in 6 subjects, most commonly dizziness (4), drowsiness (2), and headache. No clinically significant changes were observed in vital signs, ECG, or laboratory values.

## 4 Discussion

In this randomized, crossover Phase 1 trial, co-administration of PGB (80 mg) and ALA (400 mg) did not produce clinically relevant pharmacokinetic interactions in healthy adults under fasting conditions. The GMRs for Cmax and AUClast of both compounds met predefined bioequivalence criteria, and the fixed-dose combination was well tolerated. These results are consistent with prior evidence (Rhee et al., 2018) and indicate that co-formulation of PGB and ALA is pharmacokinetically feasible. While promising, these findings represent preliminary evidence that should be validated in multiple-dose studies and in patient populations.

### 4.1 Contextualizing with existing evidence

PGB remains a first-line option for PDN, though its use is frequently limited by adverse events such as dizziness and somnolence (Zaccara et al., 2011; Derry et al., 2019). Clinical trials at therapeutic doses (300–600 mg/day) have demonstrated meaningful pain reduction, but high discontinuation rates have been observed due to tolerability issues (Arezzo et al., 2008; Guan et al., 2011). ALA, in contrast, has attracted interest as a metabolic agent targeting oxidative stress and mitochondrial dysfunction in diabetic neuropathy (Mijnhout et al., 2012; Papanas and Ziegler, 2014). Intravenous ALA shows a consistent benefit, while oral formulations are hindered by high variability; however, clinical improvements have been reported at doses of 600–1800 mg/day (Hsieh et al., 2023; Mangarov et al., 2025).

Clinical trials suggest that combining these agents may improve analgesia and tolerability. Park et al. (2022) reported that low-dose PGB with ALA provided effective pain relief with fewer discontinuations, whereas the PAIN-CARE trial found no superiority of PGB with ALA over PGB alone (Gilron et al., 2024). Preclinical models support pharmacodynamic synergy, where combined administration enhances antiallodynic effects at reduced doses (Cruz-Álvarez et al., 2018; Zárate et al., 2025). Together, these findings highlight both the promise and the uncertainties surrounding the clinical utility of PGB–ALA combinations.

### 4.2 Pharmacokinetic and analytical considerations

The absence of interaction observed here aligns with previous steady-state data (Rhee et al., 2018) and reflects distinct elimination pathways: renal excretion of unchanged PGB and extensive metabolism of ALA. Lack of shared transporters or protein binding further reduces the likelihood of pharmacokinetic interference. Thus, any therapeutic benefit of combining these agents is likely to arise from pharmacodynamic rather than pharmacokinetic mechanisms.

PGB demonstrated predictable absorption and elimination consistent with published reports. In contrast, ALA exhibited high intra- and inter-individual variability, particularly in Cmax. This is consistent with its classification as a highly variable drug, which is explained by its low solubility, short half-life, and extensive first-pass metabolism (Bulut et al., 2021). Such variability may complicate dose predictability in clinical use and underscores the importance of careful design in future patient-based studies.

Administration under standardized fasting conditions minimized variability and improved internal validity; however, this design limits the generalizability of the results. Food intake has minimal impact on PGB absorption but substantially reduces ALA bioavailability (Bockbrader et al., 2010; Gleiter et al., 1996). Future studies should therefore evaluate pharmacokinetics under fed conditions to reflect real-world scenarios.

### 4.3 Clinical relevance and future directions

The safety profile in this trial was favorable, with only mild, transient adverse events, consistent with prior studies of PGB, ALA, and their combination (Rhee et al., 2018; Park et al., 2022). The rigorous crossover design, balanced male–female participation, and validated UPLC–MS/MS analysis strengthen the reliability of these findings.

Nevertheless, important limitations must be acknowledged. This was a single-dose trial in healthy adults, which does not reflect steady-state conditions or the altered pharmacokinetics seen in patients with diabetes and neuropathy. No pharmacodynamic or efficacy endpoints were included, limiting translational interpretation. Finally, inter-individual variability of ALA remains a significant challenge for clinical predictability.

Despite these limitations, the absence of significant pharmacokinetic interaction provides a foundation for further research. Future trials should include multiple-dose regimens, fed-state conditions, and patient populations, integrating pharmacodynamic and clinical outcomes. Only through such studies can the true clinical value of PGB–ALA co-formulation in PDN be determined.

## Data Availability

The original contributions presented in the study are included in the article/supplementary material, further inquiries can be directed to the corresponding authors.
